# Experimental study of reasonable mesh size of geogrid reinforced tailings

**DOI:** 10.1038/s41598-022-13980-x

**Published:** 2022-06-16

**Authors:** Changbo Du, Ben Niu, Laigui Wang, Fu Yi, Lidong Liang

**Affiliations:** 1 College of Civil Engineering, Liaoning Technical University, Fuxin, 123000 Liaoning Province China; 2 College of Mechanics and Engineering, Liaoning Technical University, Fuxin, 123000 Liaoning Province China; 3 College of Architecture and Transportation, Liaoning Technical University, Fuxin, 123000 Liaoning Province China

**Keywords:** Civil engineering, Polymers, Computational methods

## Abstract

Currently, the influence of geogrid mesh size on interface characteristics are disregarded in various codes and standards. To explore the reasonable mesh size of geogrid used for reinforced tailings, the direct shear test and pull-out test of geogrid reinforced tailings with different mesh sizes were done. The results show that the shear surface of geogrid reinforced tailings is characterized by the combined action of geogrid-tailings interface and tailings-tailings interface; the geogrid-tailings interface friction was separated from the comprehensive interface friction to analyze the influence of area ratio on it under different test conditions; and the mesh size of geogrid reinforced tailings, that is, the area ratio of the geogrid-tailings interface to the shear surface (α), has a greater influence on the pseudo-cohesion and less on the pseudo-friction angle. The friction strength of the geogrid-tailings interface increases slightly with increasing mesh size, then decreases sharply, and the reinforcement effect of geogrid quickly disappears. Considering the direct shear test and pull-out test, the reasonable mesh size of geogrid reinforced tailings should be the mesh size corresponding to α 0.47–0.55. With the increase α, the effect of the geogrid reinforced tailings can be divided into four stages where the third stage ($0.4 \le \alpha < 0.6$) is the stage with the best reinforcement effect.

## Introduction

In recent years, geogrid has been widely used in many reinforced structures such as subgrade, retaining wall, bank, slope and embankment because of its unique surface structures such as mesh and rib, which can play the role of inlay and bite. It has played a role in enhancing the strength and stability of soil. In embankment reinforcement similar to tailings dam, Arulrajah et al.^1^ conducted research on geogrid reinforced recycled foamed glass, showing that geogrid reinforcement has important engineering significance in improving embankment stability; As one of the critical structures in mine production, scholars have great concerns about the stability of tailings dam^2^, so geogrid reinforcement also has many applications and studies in enhancing the stability of tailings dam^3,4^. With the gradual application of geogrid, the interface interaction characteristics between geogrid and filler began to be gradually studied^5–15^.

The interface interaction between a geogrid and filler is a key technical index^5,16–22^, because it directly determines the stability of the reinforced structure. The interface parameters (i.e., the interface strength index of pseudo-cohesion and the pseudo-friction angle and the pseudo-friction coefficient) are the most important parameters for the design and analysis of reinforced structures^23,24^. The interface parameters of reinforced soil are mainly obtained from the direct shear test and pull-out test, and then the interface interaction characteristics of reinforced soil are analysed. Because of the different test mechanisms, the results of these two tests are quite different. Some scholars in China and abroad have compared and studied the interface interactions between geogrids and fillers^25–28^. However, scholars have not considered the unique mesh structure of geogrids in their research, so the selection of geogrid mesh size in actual reinforcement projects is still largely artificial. To solve this problem, Tang et al.^29^ believed that the interface action of the reinforced body is composed of the comprehensive friction action of the geogrid-soil interface and soil-soil interface. The friction action of geogrid-soil interface should be separated from the comprehensive friction action of interface. The geogrid-soil interface friction action should be used to characterize the reinforcement effect of geogrid to accurately describe the influence of mesh size on the reinforcement effect of geogrid. In applying geogrid reinforced tailings, the research on the interface friction characteristics of geogrid reinforced tailings has also been carried out, and the effect of mesh size on the geogrid-tailings interface has also not been considered.

This study deduces the calculation method of interface strength index, which separates the friction effect of geogrid-tailings interface from the comprehensive friction effect of interface in reinforced tailings engineering, the interface friction characteristics between geogrid and tailings with different mesh sizes were studied by indoor direct shear and pull-out tests. The effect of geogrid mesh size on the interface strength index under the two test conditions was obtained. Then, the effect of direct shearing and pull-out test on the selection of reasonable mesh size of geogrid is discussed. Then the reasonable mesh size of geogrid reinforced tailings is explored, which provided support for the design of reinforced tailings dam in practical engineering and filled the research gap in the selection of mesh size of geogrid reinforced tailings.

## Test of interface characteristics of geogrids with different mesh sizes

### Direct shear and pull-out test device design

**Figure 1:**
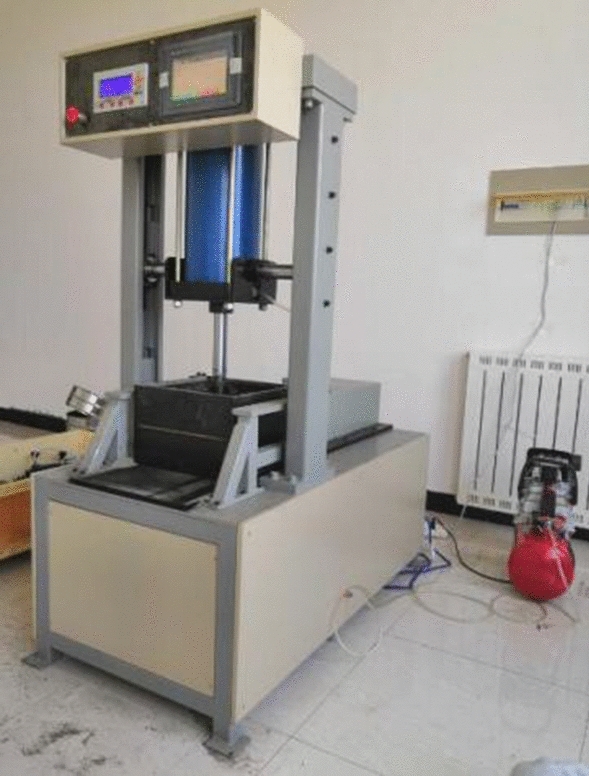
Test device.

The equipment used in this study is a patented equipment reformed from YT1200 geosynthetics direct shear pull-out test system (Nanjing Huade instrument company), which solves the shortcomings of the existing equipment. The system consists of a test box (direct shear and pull-out), a vertical loading system, a horizontal loading system, and a data acquisition system. The entire test equipment except the image recording system is installed on a test bench, so that the force of the tension and compression motor acting on the test box and the reaction force generated by the reaction device in the test box cancel each other, which is convenient for test control and reduces test errors. The test equipment is shown in Fig. 1.

### Test box

**Figure 2:**
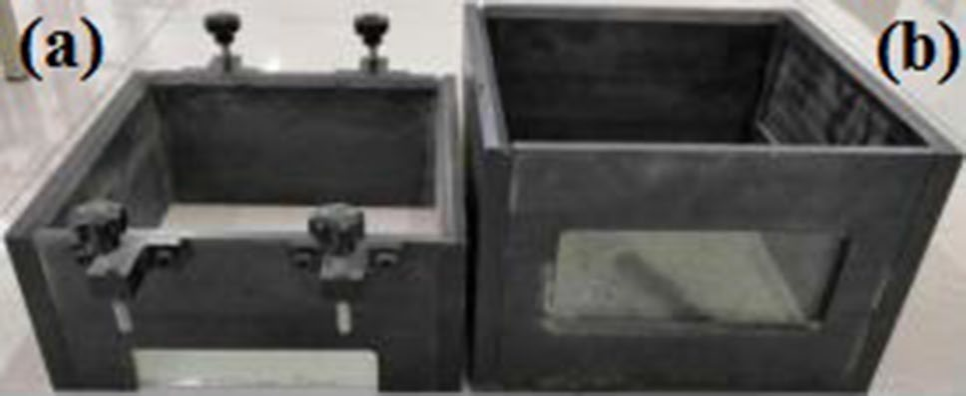
Test case.

There are two kinds of test boxes designed based on this instrument: direct shear and pull-out test boxes, as shown in Fig. 2. The direct shear test box (Fig. 2a) is divided into an upper direct shear box and a lower direct shear trolley, where the inner dimensions of the upper direct shear box are 300 × 300 × 150 mm (length × width × height). The inner dimensions of the pull-out test box (Fig. 2b) are 300 × 300 × 220 mm. A narrow slit with dimensions of 300 × 10 mm was opened in the middle of the front and back of the test groove to extract the geosynthetics. A 10 mm thick tempered plexiglass is glued inside the openings of the direct shear test box and the pull-out test box to facilitate the observation of the deformation of the reinforcement during the test and to take photos to realize the visualization of the interface between the reinforcement and soil during the test. The two test boxes of this equipment are generally larger than similar test boxes, and have a certain reduction in size and boundary effects. The bottom area is the same, which can facilitate the direct shear and pull-out tests under different working conditions, and the test results are analyzed and compared.

### Vertical loading system

The cylinder with a pressure sensor applies the overburden pressure through the reaction device in the vertical loading system. The cylinder is a 30 L air compressor. On top of the pressure loading system, a pressure plate with dimensions of 295 × 295 × 10 mm can evenly apply overlying pressures within the range of 0–200 kPa. The pneumatic loading system is used to control the vertical loading of the test equipment, which is convenient for the control and unloading of the load and can apply different constant overlying pressures to the filler in the test box.

### Horizontal loading system

The tension and compression motor of the horizontal loading system with a tension sensor has a controllable rate, which can exert a constant loading speed in the range of 0–5 mm/min and measure the test force.

### Data acquisition system

This testing machine is equipped with a control panel (see Fig. 1). The left control panel is connected to the vertical loading system to set the overlying pressure, and the right control panel is connected to the horizontal loading system to reflect the test results in real-time. On the display screen, real-time monitoring of the test data is realized so that the test can be analysed or stopped in time. When problems occur, ensuring the high efficiency of the test, the data is automatically collected and saved during the test, the test machine is connected to the computer, and the data can be exported after the test is over to the computer, the accuracy of the results is guaranteed.

### Test filling and geogrid parameter indices

The tailings filler used in the test came from the Fengshuigou tailings pond of Qidashan concentrator of Angang Mining Group, with a density of 1.83 g/cm^3^ and a moisture content of 3.75%. The physical properties of the tailings are as follows: effective particle size *d*_10_ = 0.10 mm, median particle size *d*_30_ = 0.19 mm, and restricted particle size *d*_60_ = 0.30 mm. The particle size distribution of the tailings is shown in Table 1. The calculations showed the tailings unevenness coefficient *C*_u_ was 3.5 and the curvature coefficient *C*_c_ was 1.2. The latter value is between 1 and 3, which indicates that the tailings were of poor gradation.

**Table 1:** Grain gradation of tailings.

Particle size /mm	2.36–1.18 mm	1.18–0.6 mm	0.60–0.30 mm	0.30–0.15 mm	0.15–0.075 mm	< 0.075 mm
Grading sieve residue /%	1.19	6.81	30.89	42.98	12.98	5.15
Accumulated sieve residue /%	100	98.81	92	61.11	18.13	5.15

The geogrids used in the test were glass fibre biaxial tensile geogrids (EGA30), applied in various reinforcement engineering environments with superior results. The specific material performance parameters of the geogrids are summarized in Table 2.

**Table 2:** Technological parameters of geogrids.

EGA30 geogrid	Technical index
Mesh size (length × width) /mm	12.7 × 12.7
Fracture strength /(kN m^-1^)	
Radial	30
Zonal	30
Elongation at break ≥ /%	
Radial	4
Zonal	4
Temperature resistance ≥ /℃	-100–280

### Test scheme and steps

**Figure 3:**
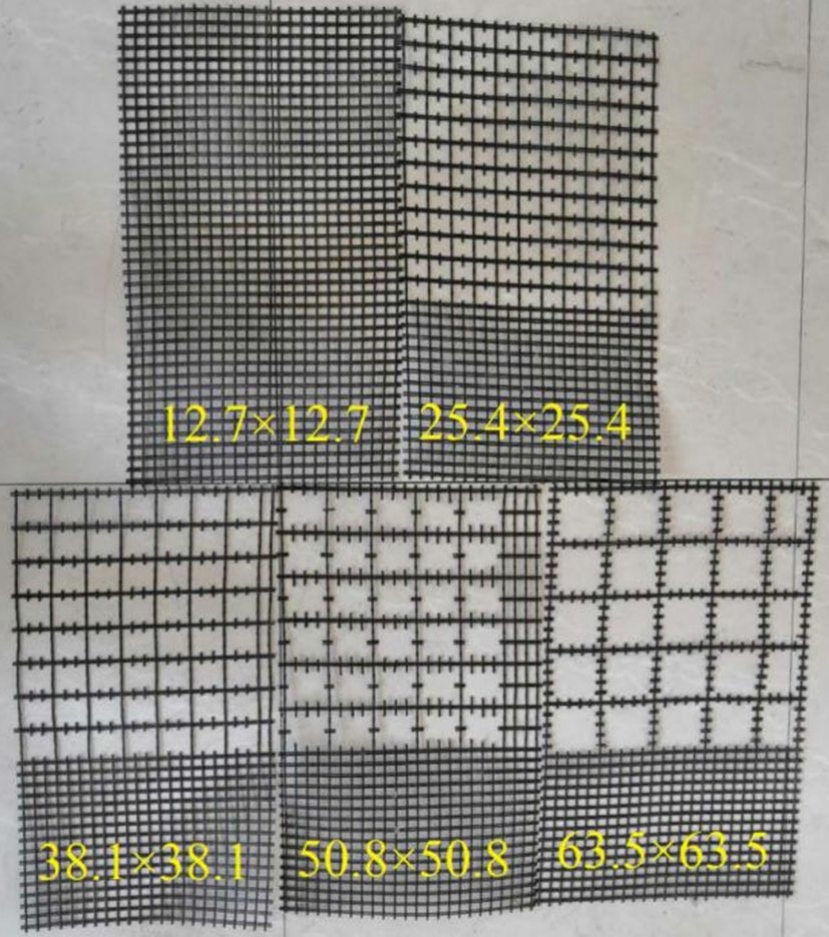
Geogrids with different mesh sizes after cutting.

Cut the geogrid used in the test according to different mesh sizes, the original size is 12.7 × 12.7 mm, and continue to cut it to other sizes in Table 3 (as shown in Fig. 3). Since the tensile strength of the geogrid will decrease after cutting, to reduce the impact of this situation on the test results, the direct shear test and pull-out test are carried out under four low-level normal stresses (10 kPa, 20 kPa, 30 kPa and 40 kPa), and five groups of test schemes are designed according to the cut geogrids with different mesh sizes, with a total of 40 groups of test schemes. Each group possessed 1–3 groups tested in parallel to reduce the discreteness of results. Table 3 shows the measured data and α of geogrids with different mesh sizes. (see below for the relevant formula in the table).

**Table 3:** Mesh size and corresponding data of geogrid under different test schemes.

Test scheme	Mesh size (length × width) /mm	Width of vertical and horizontal geogrid strip /mm	Geogrid-tailings contact area $A{}_{{{\text{geogrid}}-{\text{tailings }}}}$ / m^2^	Tailings-tailings contact area $A{}_{{{\text{tailings}}-{\text{tailings}}}}$ / m^2^	Areas ratio of the geogrid-tailings interface to the shear surface $\alpha = A\,_{{{\text{geogrid}}-{\text{tailings}}}} /A\,_{{{\text{interface}}}}$
1	12.7 × 12.7	8	0.0775	0.0125	0.8611
2	25.4 × 25.4		0.0484	0.0416	0.5378
3	38.1 × 38.1		0.0343	0.0557	0.3811
4	50.8 × 50.8		0.0265	0.0635	0.2944
5	63.5 × 63.5		0.0203	0.0697	0.2256

During the tests, the density of the tailings sand was used to control the amount of sand in the test box, and the sand loading process was stratified-compacted to ensure that each group had the same density. We applied lubricating oil evenly on both sides of the pull-out test box to reduce the size effect during testing. The speed of both the direct shear and pull-out tests was set to 2 mm/min. After the tests, the peak values of each group were recorded for subsequent analysis.

### Analysis of test results

**Figure 4:**
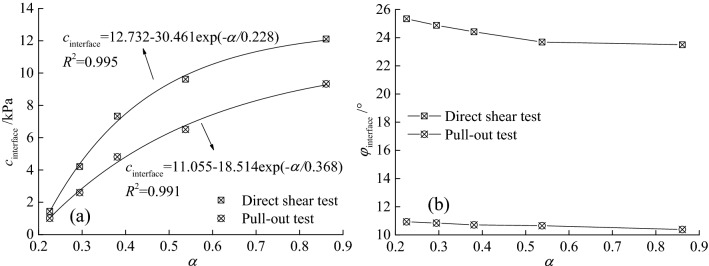
Relationship between geogrid mesh size and interface strength index during direct shear test: (**a**) pseudo-cohesion and (**b**) pseudo-friction angle.

According to the direct shear and pull-out test data of geogrid reinforced tailings with different mesh sizes of the author^30^, the interface strength indices (pseudo-cohesion and pseudo-friction angle) of the geogrid-tailings were obtained from the Mohr–Coulomb strength criterion, as shown in Fig. 4. With increasing geogrid mesh size, that is, α decreases, the pseudo-cohesion interface strength index increases, and the pseudo-friction angle does not change much. When the geogrid mesh size was increased from 12.7 × 12.7 to 63.5 × 63.5 mm, under direct shear test conditions: the pseudo-cohesion decreased from 12.11 to 1.44 kPa, which is an 88% reduction, the pseudo-friction angle increased from 23.50° to 25.34°, which is a 7.8% increase; Under pull-out test conditions: the pseudo-cohesion decreases from 9.33 to 1.01 kPa, which is an 89.2% reduction, and the pseudo-friction angle increases from 10.38° to 10.93°, which is a 5.3% increase. This is because the increase of the mesh size of the geogrid leads to the increase of the tailings-tailings contact area in the geogrid-tailings interface, which enhances the bite and inlay of the reinforcement. Therefore, under the two test conditions, the variation of geogrid mesh size significantly affects the pseudo-cohesion of interface strength index, and the influence on the pseudo-friction angle can be ignored.

### Derivation of friction strength of geogrid-tailings interface

The direct shear and pull-out tests were conducted under different normal stresses, and the fitting line conforms to the Mohr–Coulomb law. The effect of the reinforced interface is characterized by the interaction between the tailings-tailings interface and the geogrid-tailings interface. Therefore, the failures of the tailings-tailings and geogrid-tailings interfaces also conform to the Mohr–Coulomb failure criterion:

1 $$\left\{ \begin{gathered} \tau {}_{{{\text{Interface}}}} = c{}_{{{\text{Interface}}}} + \sigma {}_{{{\text{Interface}}}}\tan \phi {}_{{{\text{Interface}}}}\quad \quad \quad \quad \quad \quad \;\quad \,\,\;\;\,{\kern 1pt} (1{\text{a}}) \hfill \\ \tau_{{{\text{tailings}}-{\text{tailings}}}} = c_{{{\text{tailings}}-{\text{tailings}}}} + \sigma_{{{\text{tailings}}-{\text{tailings}}}} \tan \phi_{{{\text{tailings}}-{\text{tailings}}}} \quad \quad (1{\text{b}}) \hfill \\ \tau_{{{\text{geogrid}}-{\text{tailings}}}} = c_{{{\text{geogrid}}-{\text{tailings}}}} + \sigma_{{{\text{geogrid}}-{\text{tailings}}}} \tan \phi_{{{\text{geogrid}}-{\text{tailings}}}} \;{\kern 1pt} {\kern 1pt} {\kern 1pt} (1{\text{c}}) \hfill \\ \end{gathered} \right.$$

where $(\tau \,,\sigma )$ denote the interface friction strength and the corresponding normal stress, (kPa, kPa); $(c,\phi )$ denote the interface strength index of pseudo-cohesion and pseudo-friction angle, (kPa, °).

The resultant interface shear stress is equal to the product of the shear stress and shear area, and the following equation can be obtained under direct shear and drawing conditions:

2 $$\tau \,{}_{{{\text{interface}}}}A{}_{{{\text{interface}}}} = \tau {}_{{{\text{tailings}}-{\text{tailings}}}}A{}_{{{\text{tailings}}-{\text{tailings}}}} + \tau_{{{\text{geogrid}}-{\text{tailings}}}} A_{{{\text{geogrid}}-{\text{tailings}}}}$$

where $A{}_{{{\text{interface}}}} = A{}_{{{\text{tailings}}-{\text{tailings}}}} + A_{{{\text{geogrid}}-{\text{tailings}}}}$. $A{\kern 1pt} {}_{{{\text{interface}}}}$ denotes the area of the geogrid embedded in the direct shear or pull-out test box, which is the area of the shear plane in the test process, $A{}_{{\,{\text{interface}}}} = {0}{\text{.09 m}}^{{2}}$. $A{\kern 1pt} {}_{{\text{geogrid-tailings}}}$ denotes geogrid-tailings contact area; $A{\kern 1pt} {}_{{\text{tailings-tailings}}}$ denotes tailings-tailings contact area.

Because the composite reinforcement interface, tailings-tailings interface, and geogrid-tailings interface all produce the same action surface:

3 $$\sigma_{{{\text{interface}}}} = \sigma_{{{\text{tailings}}-{\text{tailings}}}} = \sigma_{{{\text{geogrid}}-{\text{tailings}}}}$$

According to the previous paper test results, the change of geogrid mesh size has a greater impact on the pseudo-cohesion in the interface strength index and less on the pseudo-friction angle. This study assumes that the value of the pseudo-friction angle does not change with the change of the mesh size of the reinforced tailings geogrid:

4 $$\phi \,{}_{{{\text{interface}}}} = \phi_{{{\text{tailings}}-{\text{tailings}}}} = \phi_{{{\text{geogrid}}-{\text{tailings}}}}$$

Comprehensive Eqs. (1)–(4) available

5 $$c{}_{{{\text{interface}}}} = (c_{{{\text{geogrid-tailings}}}} - c_{{{\text{tailings}}-{\text{tailings}}}} )A_{{{\text{geogrid}}-{\text{tailings}}}} /A_{{{\text{interface}}}} + c_{{{\text{tailings}}-{\text{tailings}}}} .$$

where,$A_{{{\text{geogrid-tailings}}}} /A_{{{\text{interface}}}} = \alpha$. $\alpha$ denotes the area ratio of the geogrid-tailings interface to the shear surface. $c_{{{\text{geogrid-tailings}}}}$ denotes the pseudo-cohesion of geogrid-tailings interface; $c_{{{\text{tailings}}-{\text{tailings}}}}$ denotes the pseudo-cohesion of tailings-tailings interface, default is 1 kPa, i.e.

6 $$c{}_{{{\text{interface}}}} = (c_{{{\text{geogrid-tailings}}}} - 1)\alpha + 1$$

Combining Eq. (6), Eqs. (1a), and (1c), the relationship between the friction strength of geogrid-tailings interface ($\tau_{{{\text{geogrid-tailings}}}}$) and the area ratio of the geogrid-tailings interface to the shear surface ($\alpha$) under different normal stresses can be obtained, as follows:

7 $$\tau_{{{\text{geogrid-tailings}}}} = (c_{{{\text{interface}}}} - 1)/\alpha + 1 + \sigma {\text{tan}}\phi$$

To sum up, Eq. (7) is the geogrid-tailings interface friction strength that separates the geogrid-tailings interface friction from the comprehensive interface friction. An accurate and reasonable mesh size can be obtained by analyzing the friction strength of the geogrid-tailings interface under different test conditions.

### Study of reasonable mesh size of geogrid reinforced tailings

Under the two test conditions of direct shear and pull-out, with the increase of the area ratio of the geogrid-tailings interface to the shear surface, the comprehensive interface strength index pseudo-cohesion increases in a negative index. The fitting formula is shown in the following Eq. (8). According to Eq. (8), when α = 0.22, the pseudo-cohesion of the comprehensive interface strength index under the direct shear and pull-out test conditions are 1.13 kPa and 0.87 kPa, respectively. The geogrid-tailings interface is 1.57 kPa and 0.42 kPa, respectively. Indicating that the effect of geogrid reinforced tailings has just begun to take effect; when α  = 1, that is, when the geogrid is full of tailings, the pseudo-cohesion of the comprehensive interface strength index under the two tests conditions are 12.36 kPa and 9.77 kPa. It can be seen from Eq. (6) that the value of the comprehensive interface pseudo-cohesion is the same as that of the geogrid-tailings interface.

8a $$c{\kern 1pt}_{{{\text{interface}}}} = 12.732 - 30.461\exp ( - \alpha /0.228)$$

8b $$c{\kern 1pt}_{{{\text{interface}}}} = 11.055 - 18.514\exp ( - \alpha /0.368)$$

Under normal circumstances, with the increase of α, the pseudo-cohesion of the comprehensive interface also increases, and reaches the maximum when α  = 1. But whether the reinforcement effect of the geogrid is maximized at this time, the following analysis is needed. Substituting Eq. (8) into Eq. (7), the relationship $\tau_{{\text{geogrid-tailings}}} - \alpha$ under two test conditions (The pseudo-friction angle in the following under the direct shear test and the pull-out test is the average value of multiple sets of tests, which are 24.366° and 10.702°), as shown in Eq. (9) .

9a $$\tau_{{\text{geogrid-tailings}}} = \frac{{11.{732} - 30.{461}\exp ( - \alpha /0.22{8})}}{\alpha } + 1 + \sigma \tan \varphi$$

9b $$\tau_{{\text{geogrid-tailings}}} = \frac{{{10}.{0}5{5} - 1{8}.{514}\exp ( - \alpha /0.3{6}8)}}{\alpha } + 1 + \sigma \tan \varphi$$

**Figure 5:**
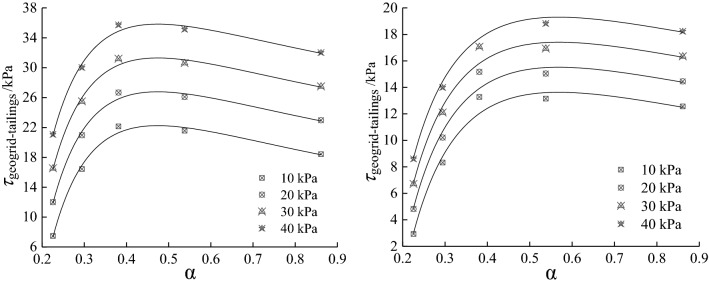
$\tau_{{\text{geogrid-tailings}}} - \alpha$ curve under different normal stress: (**a**) Direct shear test and (**b**) Pull out test.

Draw the $\tau_{{\text{geogrid-tailings}}} - \alpha$ curve under different test conditions according to Eq. (9) as shown in Fig. 5. The trend of the $\tau_{{\text{geogrid-tailings}}} - \alpha$ curve under the two test conditions is the same. As $\alpha$ increases, $\tau_{{\text{geogrid-tailings}}}$ first rises rapidly and then decreases slowly; under the same normal stress, when the geogrid mesh size is 12.7 × 12.7 mm, The $\tau_{{\text{geogrid-tailings}}}$ obtained by the direct shear test is 31.9% larger than that of the pull-out test. When the geogrid mesh size is 63.5 × 63.5 mm, the difference between the two is 60.6%. With the increase of the geogrid mesh size, the direct shear test is the more significant the difference $\tau_{{\text{geogrid-tailings}}}$ obtained from the pull-out test; this is due to the difference between the direct shear and pull-out test mechanisms. In the reinforced tailings project, the actual situation should be fully considered in the reinforced tailings position. Reasonably judge whether it belongs to direct shear friction or drawing friction to select the appropriate test method and interface parameter index.

To find the reasonable mesh size of direct shear test and pull-out test under different normal stresses, the Eq. (9) is derived to obtain the following Eq. (10). Its clear from Eq. (10) that under the conditions of direct shear or pull-out test, the curve $\tau_{{\text{geogrid-tailings}}}^{\prime } - \alpha$ has nothing to do with the normal stress, that is, the curve $\tau_{{\text{geogrid-tailings}}}^{\prime } - \alpha$ of different normal stresses is the same. Therefore, according to the derivative method, when $\tau_{{\text{geogrid-tailings}}}^{\prime } { = 0}$, the corresponding geogrid-tailings interface friction strength reaches the maximum value, and the ideal reasonable mesh size with reinforcement effect can be obtained.

10a $$\tau_{{\text{geogrid-tailings}}}^{\prime } = \exp ( - \frac{\alpha }{{0.22{8}}})(\frac{{30.{461}}}{{\alpha^{2} }} + \frac{{13{3}.{60}}}{\alpha }) - \frac{{11.{732}}}{{\alpha^{2} }}$$

10b $$\tau_{{\text{geogrid-tailings}}}^{\prime } = \exp ( - \frac{\alpha }{{0.3{6}8}})(\frac{{1{8}.{514}}}{{\alpha^{2} }} + \frac{{5{0}.{310}}}{\alpha }) - \frac{{{10}.{0}5{5}}}{{\alpha^{2} }}$$

**Figure 6:**
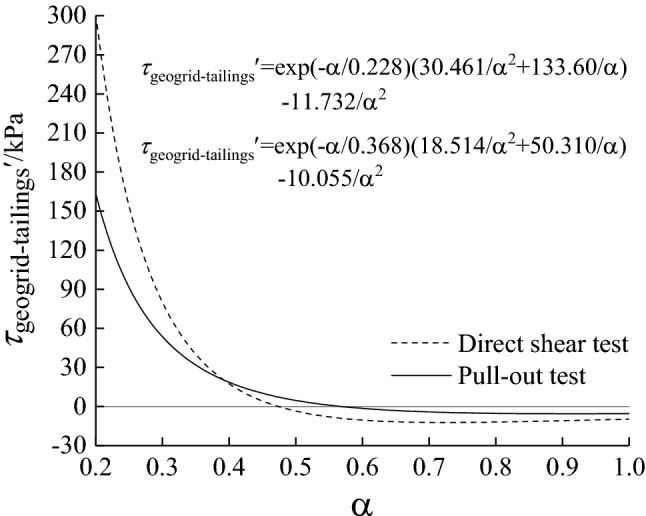
$\tau_{{\text{geogrid-tailings}}}^{\prime } - \alpha$ curve under different normal stress and different test conditions.

According to Eq. (10), the derivative of the friction strength equation of the geogrid-tailings interface is shown in Fig. 6. Under the direct shear test conditions, when α is 0.47, $\tau_{{\text{geogrid-tailings}}}$ reaches the maximum, and $\tau_{{\text{geogrid-tailings}}}$ reaches the maximum when α is 0.55 under the conditions of the pull-out test. Considering the direct shear test, pull-out test, and different normal stresses, the reasonable mesh size of the geogrid reinforced tailings corresponds to a value range of 0.47—0.55, which is the reasonable mesh of the geogrid in this test the size is 25.4 × 25.4 mm.

**Figure 7:**
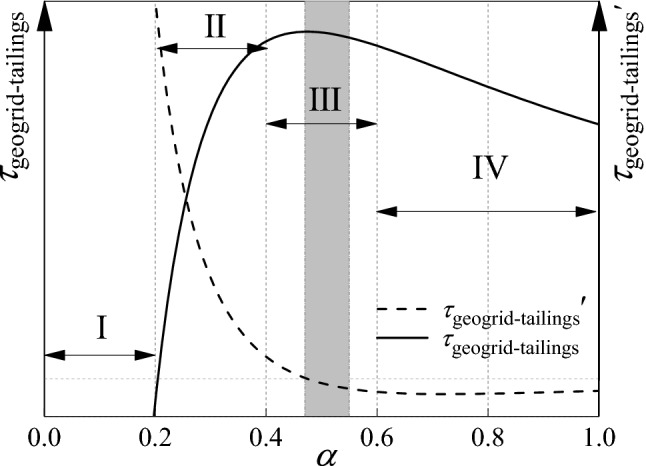
The schematic diagram of effect change of geogrid reinforced tailings.

Based on Tang's research on the reasonable mesh size of geogrid-reinforced soil^29^, this study conducts a more detailed study on the application of geogrid reinforced tailings and proposes a more accurate range of reasonable mesh size. And thus the reinforcement effect of geogrid is divided into four stages (see Fig. 7): In the I stage $0 \le \alpha < 0.2$, the effect of the geogrid reinforced tailings cannot be reflected in this stage. In the II stage $0.2 \le \alpha < 0.4$, the geogrid reinforced tailings played a role in this stage, and the reinforcement effect increased rapidly; In the III stage $0.4 \le \alpha < 0.6$, the effect of the geogrid reinforced tailings is in the transitional stage between the accelerated increase stage and the stable linear decrease stage. The shadowed part has the best reinforcement effect. In actual engineering, whether direct shear friction plays a leading role or drawing friction plays a leading role, α corresponding to the selected geogrid mesh size is recommended to be in the shaded area. In the IV stage $0.6 \le \alpha \le 1.0$, the effect of geogrid reinforced tailings shows stable linear decrease change.

## Conclusion

(1) The mesh size of geogrid reinforced tailings, the area ratio of the geogrid-tailings interface to the shear surface, has a significant effect on the pseudo-cohesion of geogrid-tailings interface strength index, and has negligible effect on the pseudo-friction angle. To analyse the effect of geogrid reinforced tailings more accurately, the relationship between the friction strength of the geogrid-tailings interface and the area ratio of the geogrid-tailings interface to the shear surface is deduced the reasonable mesh size is obtained.

(2) With the increase of the area ratio of the geogrid-tailings interface to the shear surface, the friction strength of the geogrid-tailings interface first increases rapidly and then decreases slowly. The selection of reasonable mesh size of geogrid reinforced tailings should control the area ratio of the geogrid-tailings interface to the shear surface between 0.47–0.55, within this range, the inlay and bite function of the transverse rib of the geogrid can be brought into full play, and the reinforcement effect of the geogrid is the best.

(3) The result of geogrid reinforced tailings can be divided into four stages: The third stage ($0.4 \le \alpha < 0.6$) is the transition stage between the accelerated increase stage and the stable linear reduction stage of the geogrid reinforced tailings effect. In this stage, the friction strength of the geogrid-tailings interface is greater than other stages, which is the stage with the best reinforcement effect.

## Data Availability

All data, models, and code generated or used during the study appear in the submitted article.
